# Economic value and clinical role of minimally invasive, biopsy‐based diagnostics in non‐small cell lung cancer

**DOI:** 10.1002/cncy.70152

**Published:** 2026-09-08

**Authors:** Jiayuan Guo, Bo Franzén, Rolf Lewensohn, Martin Hysek, Linus Jönsson

**Affiliations:** ^1^ Department of Neurobiology, Care Sciences, and Society Karolinska Institute Stockholm Sweden; ^2^ Department of Oncology–Pathology Karolinska Institute Stockholm Sweden; ^3^ Department of Pathology and Cancer Diagnostics Karolinska University Hospital Stockholm Sweden

**Keywords:** cost‐effectiveness, fine‐needle aspiration, health economics, non‐small cell lung cancer (NSCLC), precision medicine, rapid on‐site evaluation (ROSE)

## Abstract

**Background:**

Precision oncology requires diagnostic sampling that delivers molecularly actionable material quickly and safely. In non‐small cell lung cancer (NSCLC), core‐needle biopsy (CNB) remains common. Yet fine‐needle aspiration (FNA) has re‐emerged with rapid on‐site evaluation (ROSE), optimized cell blocks, and next‐generation sequencing. The objective of this study was to compare the economic costs of FNA versus CNB with respect to an *FNA‐first with predefined CNB escalation* strategy for NSCLC clinical pathways.

**Method:**

The authors synthesized evidence on diagnostic adequacy, complication rates, and molecular performance of cytology versus core tissue. A microcosting framework decomposed total diagnostic episode costs into sampling, processing, complications, and remedial procedures. This framework was applied to Sweden’s Standardized Cancer Care Pathway for NSCLC using decision‐tree modeling and sensitivity analyses.

**Results:**

Modern cytology workflows with ROSE achieve a nondiagnostic sampling rate ≤6% and support programmed death‐ligand 1 and broad next‐generation sequencing with high mutation concordance to surgical specimens. Compared with CNB, FNA incurs fewer major complications and facilitates repeat sampling. In the Swedish illustration, an FNA‐first pathway with ROSE reduced expected per‐patient diagnostic cost by approximately 33% (7748 vs. 11,637 Swedish krona). The results were robust to ±50% variation in complication rates and ±5%–10% variation in adequacy.

**Conclusions:**

Reframing *adequacy* around molecular fitness and time to actionability supports FNA where ROSE exists, with codified, same‐episode CNB escalation. This approach advances safety, capacity, and timeliness without compromising molecular yield. The authors provide a transferable cost‐consequence model and implementation checklist for health systems. Local recalibration is required for absolute cost values, and multisite calibration is encouraged to establish generalizability across the different settings.

## Abstract 2

This article highlights the economic benefits of fine‐needle aspiration versus core‐needle biopsy in transthoracic biopsies from a health‐economic perspective. With rapid on‐site evaluation, a fine‐needle aspiration saves 3889 Swedish krona per patient compared with a core‐needle biopsy, supporting fine needle aspiration as a safe, cost‐effective, first‐line diagnostic approach in precision oncology.

## INTRODUCTION

### The tissue‐acquisition crisis in the precision oncology era

In the era of precision oncology, treatment eligibility hinges on molecular stratification, making biopsy not just diagnostic but determinative for therapeutic access. Non‐small cell lung cancer (NSCLC) accounts for 85% of cases and remains a leading cause of global cancer‐related mortality. It requires timely and adequate tissue acquisition to enable molecular testing.^1^, ^2^ NSCLC is projected to increase 70% by 2050. This intensifies pressure on the molecular diagnostics infrastructure required for treatment stratification.^1^ Despite significant advances in understanding molecular pathogenesis and the development of targeted therapies and immunotherapies, real‐world registry studies indicate that the median time from radiographic suspicion to histologic diagnosis in NSCLC is approximately 20 days.^3^ Such diagnostic delays, although heterogeneous by stage, are widespread and represent a critical challenge: delivering timely precision therapies. Delays in diagnosis not only compress the window for therapeutic intervention but also compromise the feasibility of acquiring high‐quality tissue samples for next‐generation sequencing (NGS), gene expression and fusion protein analyses, and immunohistochemical (IHC) profiling, which are important for precision treatment.^4^ Core‐needle biopsy (CNB) has been favored for its perceived adequacy, histologic yield, and role in exact diagnosis. However, CNB is invasive, is associated with higher complication rates, including the risk of spreading tumor cells, and demands more specialized resources.^5^ Minimally invasive fine‐needle aspiration (FNA), once undervalued because of concerns over cellularity and architectural detail, is experiencing a resurgence. With advances in cytology, cell‐block preparation, rapid on‐site evaluation (ROSE), and NGS, FNA now meets and may exceed the molecular diagnostic thresholds necessary for NSCLC workup and longitudinal adaptation of treatment.

FNA now holds the possibility of becoming the core diagnostic tool in precision oncology. This technique was initially established as a safe, minimally invasive tumor‐diagnostic method pioneered by Sixten Franzen. It has been subsequently standardized for diagnosis in many different organ systems, for example, the Bethesda System for Reporting Thyroid Cytopathology developed in the United States^6^, ^7^ and the International Academy of Cytology, International Agency for Research on Cancer, and World Health Organization cytopathology reporting systems series for lung cytology, pancreatobiliary cytology, and lymph node cytology.^8^ It is now further developed with molecular integration. Studies have confirmed that FNA samples can reliably detect gene mutations such as *BRAF* V600E and *TERT* promoter mutations.^9^ Today, image‐guided and endoscopic techniques (such as endobronchial ultrasound‐guided FNA for lung cancer staging) have further extended it into an integrated platform that connects morphology, molecular spectrum analysis, and clinical decision‐making.^10^ The role of FNA will likely extend beyond providing a preliminary diagnosis, with a wide range of molecular panels enabling the identification of specific tumor subtypes to inform treatment decisions. This is particularly relevant for longitudinal, treatment‐oriented biomarker analysis, especially when treatment‐induced drug resistance emerges, and repeated sampling at different stages of the disease is required to dynamically adjust treatment strategies.^11^, ^12^ With its minimally invasive nature, high safety, and high compatibility with modern molecular techniques, FNA may very well meet serial monitoring requirements.^13^

This prospective review evaluates FNA and CNB across clinical, molecular, economic, and policy dimensions. Now, biopsy strategy in NSCLC could very well be restructured to prioritize safety, efficiency, and accessibility and also to enhance longitudinal molecular assessment.^14^

## MATERIALS AND METHODS

### Diagnostic modalities at a crossroad: From tissue quantity to molecular fitness

#### CNB as the *gold standard*

The majority of first‐line biopsies use CNB as a time‐honored dogma of medicine.^15^ CNB yields a core specimen that allows for extensive histologic sectioning and molecular analysis. Although the total number of slides per case is protocol‐dependent and may vary across institutions, the key differentiator is total pathology hands‐on time: in our institutional workflow, FNA requires approximately 10 minutes of technician preparation versus 60 minutes for CNB, with correspondingly fewer pathologist review minutes. This workload difference, rather than slide count alone, drives the staffing and cost advantages of FNA, which are particularly relevant given the pathologist shortage in Europe (e.g., 2.1 per 100,000 in Germany).^16^

Moreover, a proportion of initial CNB procedures fail to yield sufficient representative material for molecular profiling, necessitating a re‐biopsy, which is typically scheduled a few weeks after and delays the patient's discussion at the tumor board (multidisciplinary team) meeting and successively delays the start of therapy. This inefficiency brings the new idea for a synergistic FNA–CNB approach.

#### The resurgence of FNA

The modern revival of FNA is rooted in both technological innovation and its enduring legacy in the diagnosis of cancer. Although often perceived as a recent alternative to CNB, FNA has a rich historical lineage. Early needle biopsy techniques can date back to the 19th century, but it was only in the 1930s that American surgeons Hayes Martin and Edward Ellis pioneered its use for diagnosing palpable masses, particularly in head and neck tumors.^17^ Their contributions established FNA as a minimally invasive diagnostic tool in the early 20th century. Jumping to the mid‐20th century, Scandinavian radiologists like Nils Söderström and Joseph Zajicek refined FNA for cytological application.^18^ Later in the 1960s, Swedish cytopathologist Sixten Franzén developed a flexible spinal needle for prostate biopsies, proving that FNA can be applied to deeper tissues.^6^, ^19^ After a decade, the appearance of ultrasound‐guided and computed tomography (CT)‐guided FNA enabled precise targeting of nonpalpable lesions, like liver and pancreas, solidifying the role of FNA in cancer diagnosis.^20^ In contrast, the 1990s saw the emergence of CNB, which aimed to overcome limitations in assessing tissue architecture by retrieving core samples.^21^ Image‐guided CNB became more standardized with the advent of spring‐loaded, semi‐automated devices.^22^, ^23^

Despite the rise of CNB, recent advances in cytologic preparation, ROSE, and molecular testing have renewed interest in FNA as a diagnostic platform in precision oncology.^24^ Modern FNA samples can provide both diagnostic and molecularly adequate material, including for NGS, supporting its integration into contemporary diagnostic pathways.^25^

### Evidence synthesis

#### Clinical performance and molecular diagnostic capability

FNA and CNB occupy different positions in terms of diagnostic sufficiency and operational risk spectra. Although CNB maintains slightly higher first‐attempt adequacy (approximately 92% vs. 85%), it is worth noting that the gap significantly narrowed after the introduction of ROSE.^26^ More important, CNB has substantially higher risks of complications: pneumothorax occurs in up to 7% of patients compared with 3% with FNA.^25^, ^26^ In addition, the bleeding risk of CNB is more prominent, especially among patients receiving anticoagulants or with deep locations.^24^

From the perspective of molecular diagnostic efficacy, FNA has transcended its traditional role as a stopgap measure in cytology and developed into a reliable technical platform in precision oncology. Three attributes underpin this shift. (1) FNA can shorten the reporting cycle by 48–72 hours because it bypasses the time‐consuming decalcification and paraffin‐embedding steps required for CNB. Its direct smear technique does not require paraffin‐embedding steps and can support key immunocytochemical assays (such as programmed death‐ligand 1 [PD‐L1] expression analysis and immunophenotyping) on the day of sampling.^27^ Meanwhile, ROSE‐guided immediate sample assessment can effectively control the nondiagnostic sample rate at ≤6%, making its tissue acquisition success rate comparable to that of CNB.^28^ (2) The low rate of major complications (approximately 0.3% with FNA vs. 1.9% with CNB) permits repeated, targeted re‐sampling.^29^ This is particularly important in clinical practice, for example, for monitoring the development of drug resistance that can occur during the treatment of patients who have epidermal growth factor receptor (EGFR)‐positive or anaplastic lymphoma kinase (ALK)‐positive NSCLC (such as the acquisition of drug‐resistant mutations like EGFR T790M or ALK G1202R.^30^ (3) Existing research has fully confirmed that FNA cell blocks prepared by modern technology can stably produce DNA at the level of ≥100 ng in the vast majority of cases (approximately 94%).^5^ This output is sufficient to support the detection of large‐scale (>300 genes) NGS panels, and it demonstrates a high degree of consistency (about 99.2%) in the detection of driver gene mutations compared with the reference results of surgically resected specimens.^5^

#### Economic evaluation

Does economic evaluation matter for biopsy choice? In most oncology pathways, diagnostic spending represents only a small portion of total costs over the longitudinal course of care, yet biopsy choice can have a disproportionate effect on time‐to‐time treatment, complications, and access to care. Therefore, economic evaluation should go beyond the unit cost of the procedure and consider the downstream consequences, including re‐biopsy cascades, pathology bottlenecks, and equity of access to the decentralized settings.

To evaluate the economic implications of adopting FNA, compared with CNB, as the first‐line diagnostic procedure in NSCLC, we structured the diagnostic workflow into discrete cost‐driving components (Table 1). This modular approach enables the total cost per procedure to be expressed as the sum of resource use across six key process domains:

C_total=C_prep+C_sampling+C_ROSE+C_pre‐staining+C_staining+C_evaluation

**TABLE 1:** Cost variable framework for modelling fine‐needle aspiration and core‐needle biopsy diagnostic workflows.

Workflow step	FNA components	Cost variable	CNB components	Cost variable
Preparation	Radiology guidance, sterile setup	C_prep	Same	C_prep
Sampling	Syringe, needle, handle, glass slides	C_sampling	CNB pistol, formalin container	C_sampling
ROSE	Quick stain, microscope	C_ROSE	—	—
Prestaining	—	—	Fixation, dehydration, paraffinization, cutting	C_prestaining
Staining	Automated	C_staining	Automated	C_staining
Evaluation	Per‐slide assessment	C_evaluation	Per‐slide assessment	C_evaluation

Abbreviations: C_, cost; CNB, core‐needle aspiration; FNA, fine‐needle aspiration; prep, preparation; ROSE, rapid on‐site evaluation.

By substituting the variables listed in Table 1 with local unit costs and resource use estimates, researchers and health systems can replicate the analysis in their own contexts, enabling comparative cost‐consequence evaluations of biopsy strategies under different scenarios.

##### Global view

Published work on biopsy economics is scarce and fragmented across different modalities. Few studies focus on micro‐costed comparisons of FNA and CNB in lung cancer with explicit modeling of re‐biopsy cascades and complication management. For the economic evaluation of FNA versus CNB, the cost‐consequence view is a straightforward method that any health system can adapt. The key is: Which modality delivers an actionable result sooner, is less invasive, and has a lower total system cost? Rather than focus on unit price or unit quality‐adjusted life‐years alone, report disaggregated outcomes, including: (1) per‐patient diagnostic cost, covering sample collection, laboratory processing, and complication management; (2) time to actionable report (first pathology [anatomic] diagnosis and, where relevant, NGS initiation; (3) adequacy and biopsy rates; and (4) basic capacity markers (slides per case and staff minutes) that capture histopathology bottlenecks. This general framing keeps methods light while still reflecting the downstream cascades of diagnostic choice (delays, re‐biopsy loops, etc.) and allows fair comparison across settings.

With this general frame established, we then move to scenario analysis that applies the same outcomes to a concrete context. Below, we use Sweden’s Standardized Cancer Care Pathway (SVF) times as a detailed example, implementing a redesigned decision tree and micro‐costing to quantify costs, time and complications.

##### Case study: Swedish (SVF) in NSCLC

###### Scope and horizon

**FIGURE 1:**
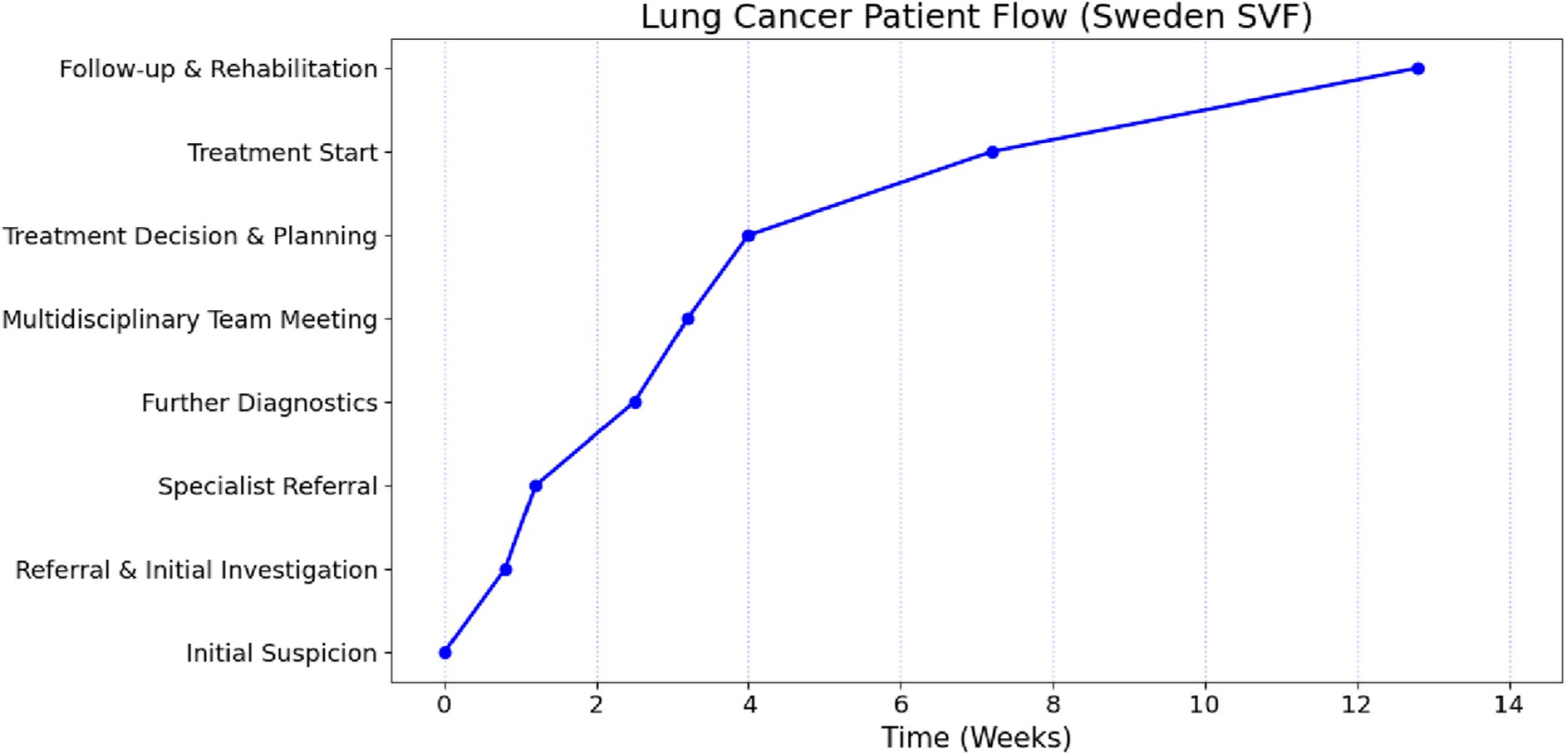
Lung cancer patient flow (Sweden SVF). CNB indicates core‐needle biopsy; FNA, fine‐needle aspiration; SVF, Sweden’s standardized Cancer Care Pathway.

This case study explores how the Swedish SVF framework implements an FNA‐first, CNB‐supplemented strategy in the diagnosis of suspected NSCLC and clearly optimizes around the modern molecular oncology workflow. The research from a health payer perspective, focusing on the initial diagnostic episode, is illustrated in Figure 1. This analysis is independent and portable. Other systems can apply their conclusions by replacing local data.

###### Diagnostic pathway

The overall process from clinical referral to the initiation of treatment is illustrated in Supporting Information S1: Figure S1. The path contains two main strategies:

- FNA priority strategy: Fine‐needle aspiration sampling is preferred, followed by ROSE. If the sample is insufficient or histological structure is required, it should be upgraded to CNB in a timely manner.
- CNB priority strategy: Core needle biopsy is preferred; if the sample collection is insufficient or complications occur, a re‐puncture (CNB or FNA) should be arranged.

The key objective of SVF is to complete the first pathology (anatomic) diagnosis and initiate molecular testing within a strict time frame, ensuring that patients can enter multidisciplinary team discussions and treatments as scheduled. Figure 1 also marks the recommended time points by regional cancer centers, highlighting the role of FNA in shortening procedural time, reducing complications, and minimizing repetitive puncture procedures.

###### Identifying workflow decision points

The choice between FNA and CNB is not binary but is sequential and complementary within the SVF. Key decision points are:

- Decision point A: Patient and lesion characteristics: Size, location, radiologic appearance of the lesion (e.g., peripheral ≥1.5 cm vs. central; solid mass vs. ground‐glass opacity), proximity to vessels, patient's anticoagulation status, and comorbidities. For purely ground‐glass opacities, FNA diagnostic accuracy is lower; thus CNB should be prioritized for such lesions. FNA is preferred for solid or mixed lesions with a safe biopsy window, particularly in higher risk locations or frail patients.
- Decision point B: Diagnostic and molecular intent: Is histologic architecture *absolutely required* (e.g., subtyping of lymphoma)? Can cytology (using smears and cell blocks) provide adequate material for *actionable* molecular biomarker testing (PD‐L1 IHC, EGFR/ALK/ROS1/KRAS, and large‐panel NGS)? For most NSCLC cases, the primary goal is obtaining sufficient tumor cell content and nucleic acid yield for molecular assays, a need effectively met by FNA.
- Decision point C: Institutional resources and capabilities: Availability of ROSE is a key enabler for the FNA‐first strategy. Other factors include expertise in cell block processing, the turnaround time for molecular tests, and the ability to schedule a complementary CNB within 24–48 hours if needed.

**FIGURE 2:**
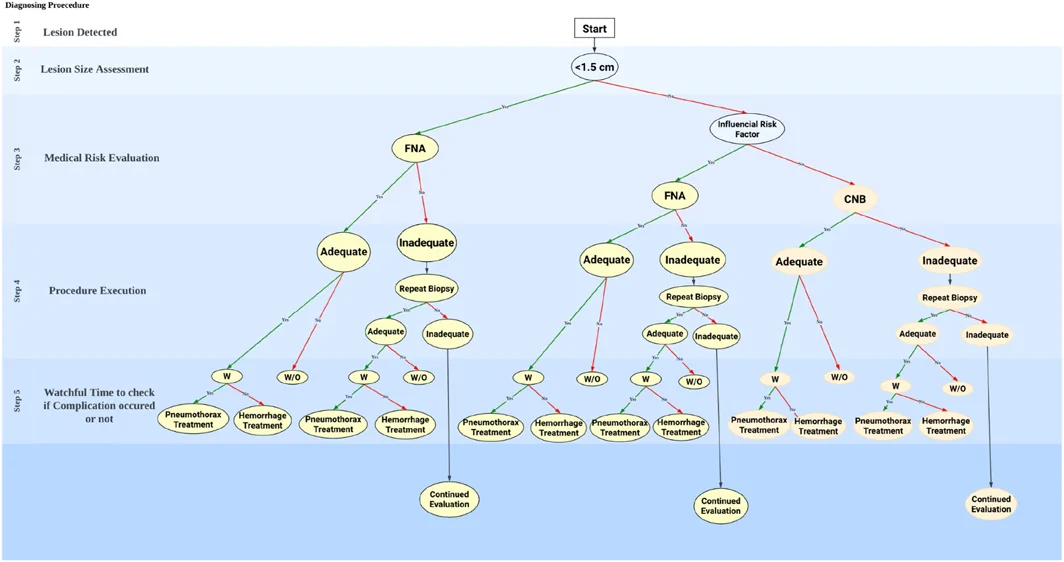
Decision tree of acquiring adequate material for diagnosing non‐small cell lung cancer. CNB indicates core‐needle biopsy; FNA, fine‐needle aspiration; W, with; W/O, without.

A practical illustration of these considerations is provided in Figure 2.

In practice, an FNA‐first approach is favored when access is safe, molecular results are urgently needed, and ROSE can confirm adequacy; whereas CNB is the preferred first step when tissue architecture is mandatory or when prior FNAs have been inconclusive. Crucially, the FNA‐first pathway should always incorporate a predefined escalation protocol to CNB; for example, if ROSE indicates that the specimen is inadequate or offers a morphologic differential diagnosis that requires histologic subtyping, the patient can transition immediately to a reserved CNB slot, thereby preserving diagnostic timelines and ensuring that molecular testing proceeds without delay.

###### Sample processing

**FIGURE 3:**
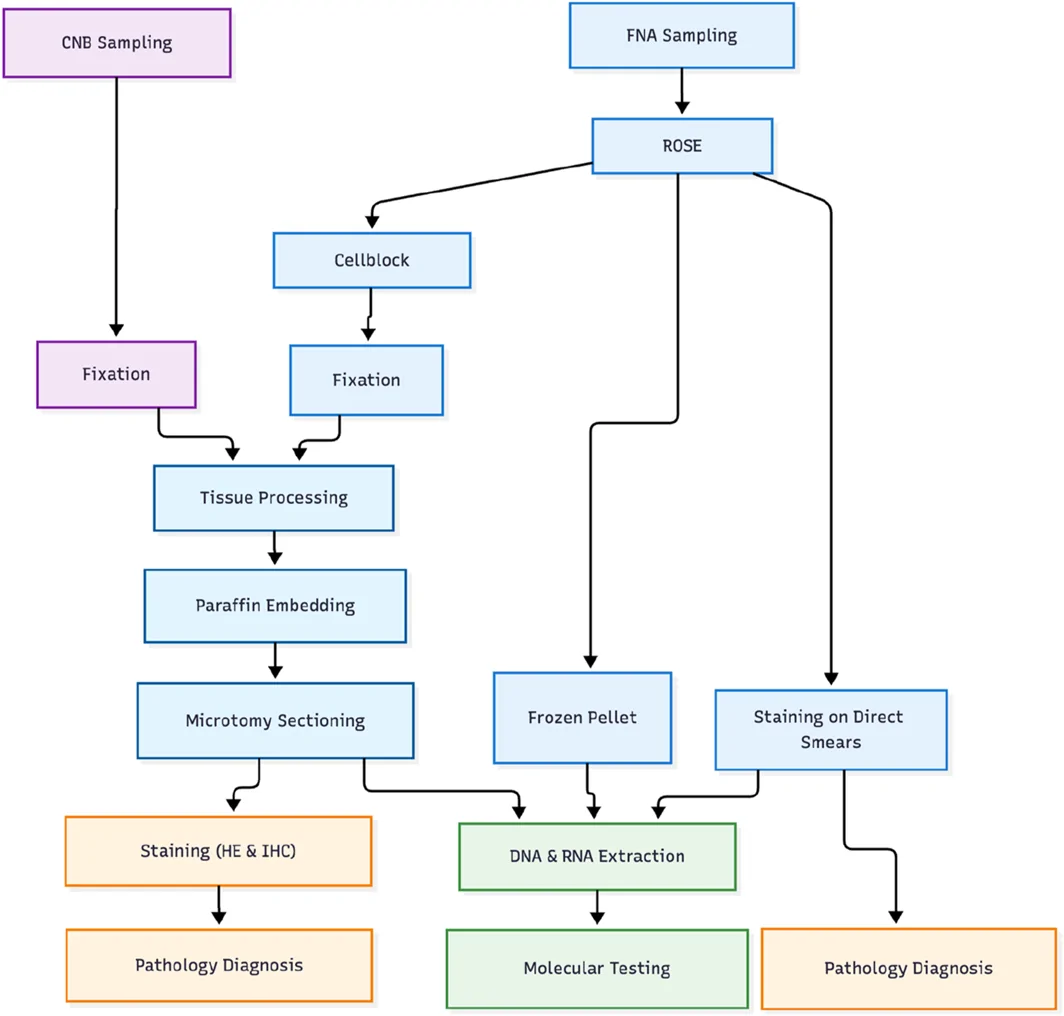
Workflow of FNA and CNB sample processing. CNB indicates core‐needle biopsy; FNA, fine‐needle aspiration; HE, hematoxylin and eosin staining; IHC, immunohistochemistry; ROSE, rapid on‐site evaluation.

The true value of a biopsy sample is determined in the lab. Thus, the sample processing pipeline is critical, as it directly impacts the sample's viability for both morphological diagnosis and advanced molecular testing. Figure 3 illustrates the parallel processing pathways for FNA and CNB samples, highlighting how both converge into an FFPE block:

###### Sample processing with economic impact

Because FNA typically produces a limited number of slides and can determine the adequacy of samples instantly through ROSE, its laboratory processing time and the human cost required for diagnosis are significantly reduced compared with CNB. In our institutional routine, the average number of slides per case is three for FNA and 12 for CNB. More important, the hands‐on laboratory time is substantially lower for FNA (approximately 10 minutes) than for CNB (approximately 60 minutes), which reduces the workload of technicians and pathologists. This efficiency advantage is reflected in the operational cost. The time investment of technicians and pathologists is clearly inclined to support FNA.

Furthermore, the follow‐up treatment related to complications also exacerbated this difference. The incidence of pneumothorax and bleeding in patients undergoing FNA is relatively low, reducing the need for additional clinical intervention measures and thus avoiding the additional sample processing and repetitive diagnosis work that is usually necessary after the occurrence of CNB‐related adverse events.

Finally, in patients without complications, the diagnostic cycle of FNA has consistently been shorter, ensuring the efficiency of the overall workflow of the SVF. However, the implementation of ROSE introduces an additional time burden because a qualified cytopathologist or trained technician must be present during the procedure to prepare and evaluate slides in real time. This dedicated commitment of personnel, which could otherwise be allocated to the review of other cases, may partly offset the apparent time advantage of FNA and thus should be considered when evaluating its overall efficiency. In conclusion, these findings clearly demonstrate how differences in sample collection and processing are passed down layer by layer, ultimately affecting the operational efficiency of the entire medical system. This confirms that the *FNA‐first* strategy not only reduces the workload of the laboratory and lowers the amount of work related to complications but also shortens the time required to obtain molecular reports that can guide treatment.

###### Cost‐benefit calculation model

This model provides a framework for gradually estimating and comparing the expected costs of the *FNA‐first* and *CNB‐first* strategies. To further validate the model structure, an illustrative Swedish example was reconstructed (see Supporting Information S1: Methods). By using hypothetical but realistic unit costs, the model estimated that an FNA‐first strategy yielded an expected diagnostic cost of 7748 Swedish krona (SEK) compared with 11,637 SEK for the CNB‐first strategy, resulting in a per‐patient saving of 3889 SEK (approximately 33%).

**FIGURE 4:**
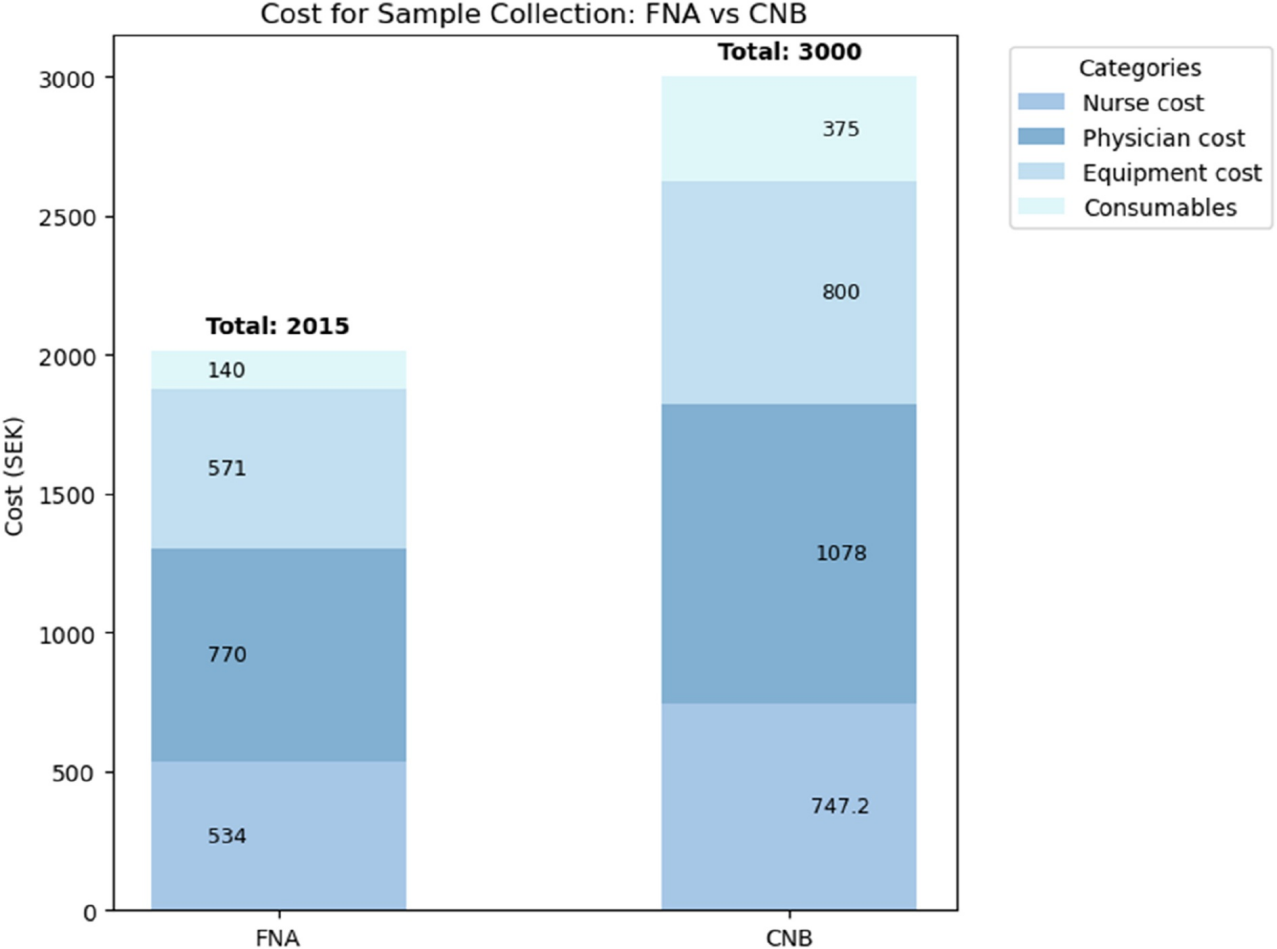
Comparison of total and component costs for FNA and CNB sample collection. CNB indicates core‐needle biopsy; FNA, fine‐needle aspiration.

**FIGURE 5:**
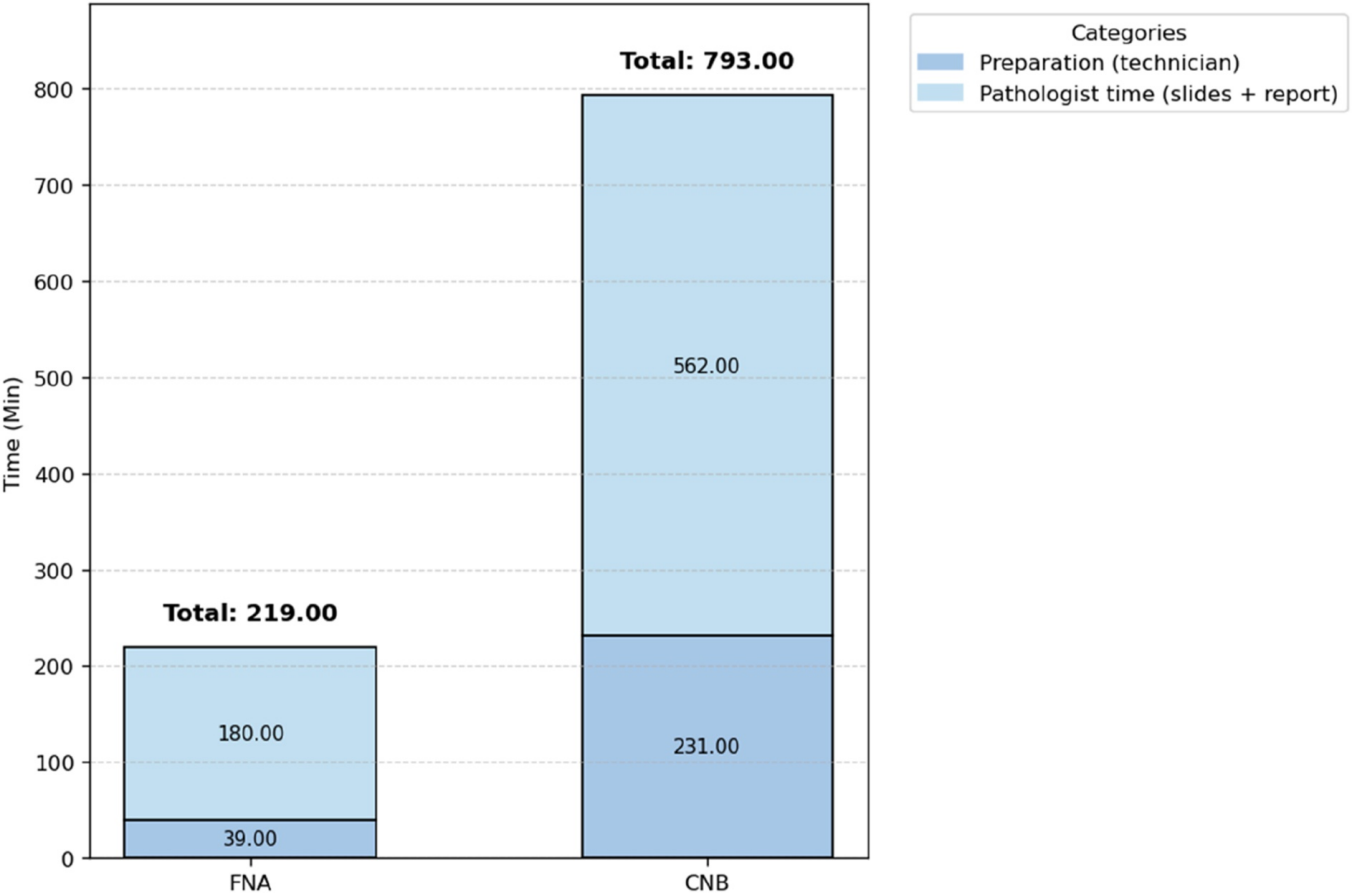
Comparison of sample processing costs for FNA and CNB procedures. CNB indicates core‐needle biopsy; FNA, fine‐needle aspiration; Min, minutes.

The cost difference is primarily explained by two drivers: sample collection and sample processing. For sample collection, CNB requires greater physician analysis time and consumables because local anesthesia and a sterile work environment are required, leading to markedly higher procedural expenses, as indicated in Figure 4. For sample processing, CNB incurs a 3.6‐fold increase in cost relative to FNA, largely because of longer technician preparation (60 vs. 10 minutes; Figure 5). Although the number of slides can vary by institutional protocol, we used total pathology labor time (minutes per case) as the primary cost driver because it directly captures technician and pathologist workload irrespective of slide‐count variations. This approach ensures that our cost estimates are generalizable across different cytologic preparation methods (e.g., smears, liquid‐based cytology, or cell blocks).

Although the absolute cost levels differ from the main analysis (2015 vs. 3000 SEK for FNA vs. CNB), the direction and magnitude of cost advantage remain consistent. Both analyses highlight the same drivers: CNB incurs substantially higher pathology costs, complication‐related expenses, and remedial procedures.

**FIGURE 6:**
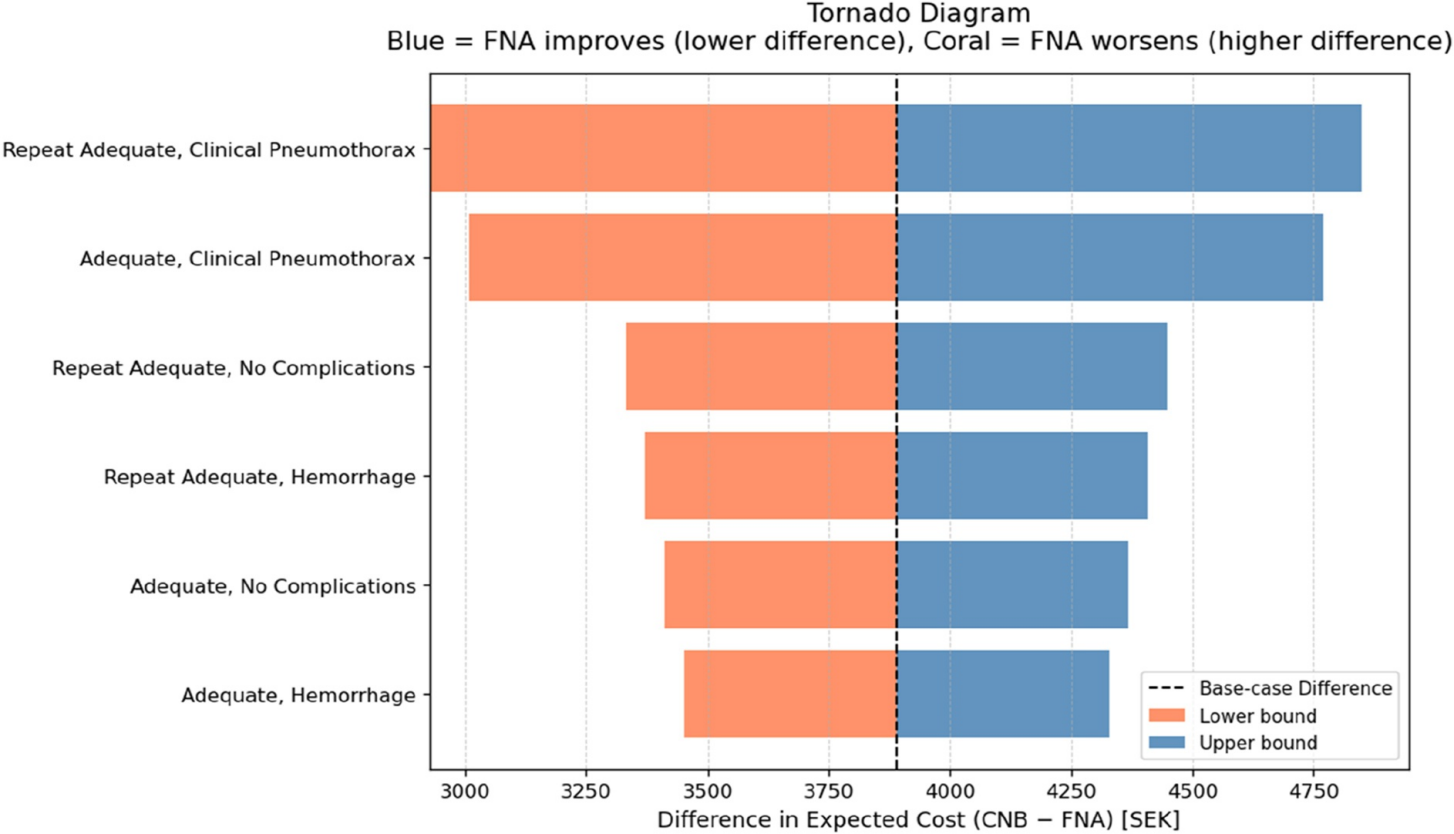
Tornado diagram of input parameter sensitivity. CNB indicates core‐needle biopsy; FNA, fine‐needle aspiration.

Sensitivity analyses across complication rates (±50%) and adequacy rates (±5%–10%) confirmed the robustness of the conclusion that FNA maintains economic advantage under plausible parameter variations, as illustrated in Figure 6. The tornado diagram displays the extent to which changes in key input parameters (e.g., complication rates, adequacy, or procedural costs) influence the incremental cost difference.

In this type of diagram, the length of each bar represents the magnitude of the parameter’s impact, with longer bars indicating stronger influence on costs. It important to note that, even for the most influential parameters, the cost of FNA remained lower than CNB. FNA consistently maintained a cost advantage, indicating robustness of the base‐case findings. No single parameter shift was sufficient to eliminate the cost difference, suggesting that the economic benefit of FNA is resilient to reasonable fluctuations in clinical input assumptions.

We also examined the hypothetical scenario in which CNB is performed with ROSE (e.g., touch imprint cytology). Although this practice exists in some institutions, adding ROSE to CNB increases personnel and consumable costs without reducing the inherent higher complication risk (pneumothorax, bleeding) of CNB itself. Under this *both with ROSE* comparison, the FNA‐first pathway retains its cost advantage, and the cost gap widens because CNB adds ROSE costs on top of its already higher baseline expenses. Therefore, the economic benefit of FNA‐first is reinforced, not weakened, by the potential use of ROSE on CNB.

###### Limitations and transferability

The results rely on local workflows and unpublished model inputs from a single institution. Findings are derived from a single institutional workflow and require local re‐parameterization before generalization to other health care settings. The transformation to other settings should re‐parameterize staffing costs, complication probabilities, and pathology capacity. Finally, long‐term health outcomes and patient‐reported measures were not included and warrant probabilistic sensitivity analysis/Markov extensions.

To conclude the Sweden case, it exemplifies how, once time, safety, and capacity are explicitly valued, FNA emerges as the value‐consistent first pass in NSCLC diagnostics. These findings position FNA as not only a clinically comparable option but also as a resilient, economically rational strategy for integration into high‐throughput oncology frameworks. The same logic can guide adoption in other systems with appropriate local calibration.

First, our analysis is based on standard CT‐guided or ultrasound‐guided percutaneous FNA. More advanced techniques, such as navigational bronchoscopy or endobronchial ultrasound, would incur additional equipment and personnel costs and may alter the cost comparison. Therefore, our findings should be interpreted as applying to the standard percutaneous setting rather than to all FNA modalities.

Second, our cost model assumes FNA as a standalone procedure without concurrent transbronchial biopsy. If additional passes or concurrent biopsies are performed, the cost advantage of FNA would diminish and should be re‐calculated with local data.

Third, our conclusions are based on centers with CT or ultrasound guidance capacity. In resource‐limited settings without such equipment, CNB may remain the more feasible option. This limits the generalizability of our findings to well equipped institutions.

## RESULTS AND DISCUSSION

### Positioning FNA and CNB

#### Suitable use cases

FNA is the preferred modality when three conditions meet: Anatomic access is safe, the clinical question demands speed to an actionable molecular result, and the care setting can rapidly confirm adequacy (e.g., ROSE) with a clear path for escalation if needed. In practice, this strategic division means that CNB is typically reserved for sampling larger, peripheral lesions of sufficient size (often >1.5 cm) with a clear imaging window that exists, and critical structures can be safely avoided. Conversely, FNA is particularly valuable for smaller, central lesions located near high‐risk anatomic structures (e.g., major vessels or airways) yet still require a comprehensive diagnostic workup. In scenarios that require rapid results for biomarkers, for example, PD‐L1 or broad NGS panels, and in clinics capable of same‐day adequacy assessment, FNA delivers immediate system benefits. The availability of ROSE further supports an FNA‐first strategy by allowing immediate adequacy assessment. Cytology that meets a pragmatic adequacy threshold (approximately ≥100 viable tumor cells or a high tumor cell fraction) can proceed directly to molecular work‐up, targeting NGS initiation within 72 hours rather than legacy week‐long pathology cycles. For patients with coagulopathy or significant comorbidity, FNA may also be preferred in for those at higher procedural risk.

Conceptually, adequacy is defined by molecular utility rather than tissue volume: For key targets, cytology achieving this threshold generally demonstrates high concordance (κ ≥ 0.85) with core tissue at substantially lower episode cost (approximately 33% in the Swedish model). CNB remains appropriate when preserved architecture is essential (e.g., suspected lymphoma) or when serial cytology remains nondiagnostic despite ROSE.

#### Systems opportunities

Despite the documented advantages of FNA, including a superior safety profile and lower complication rates, its use in the initial diagnosis of NSCLC within the Swedish SVF remains lower than anticipated.^26^ Sweden’s SVF aims to achieve rapid, fair, and uniform cancer diagnosis, setting clear time nodes for initial examinations.^31^ However, in its actual operation, this system is confronted with a fundamental policy tension: Will the pursuit of diagnostic *sufficiency* come at the expense of system efficiency?

The current SVF framework defaults to prioritizing CNB, particularly for lesions larger than 1.5 cm, to guarantee tissue volume and integrity for molecular analysis. This preference reflects a prevailing assumption that cytology specimens obtained by FNA are insufficient for comprehensive biomarker testing. However, as discussed above, accumulating evidence supports the molecular adequacy of modern cytological preparations, including FNA cell blocks.^32^, ^33^ The continued preference for CNB may therefore reflect, at least in part, an entrenched *gold‐standard* convention rather than technical feasibility alone.

This default reliance on CNB introduces systemic inefficiencies:

- Resource concentration pressure: CNB relies on specialized radiologic and pathologic infrastructure, which may concentrate diagnostic activity within tertiary centers and increase pressure on limited personnel and capacity.
- Downstream resource burden: Procedure‐related adverse events may generate additional health care use and extend the diagnostic pathway.
- Re‐biopsy–related delays: Inadequate or nonrepresentative sampling may still require repeat biopsy, delaying subsequent diagnostic and treatment decisions.

By contrast, FNA may offer greater workflow flexibility and support more efficient use of diagnostic resources. Where ROSE is available, FNA can support real‐time triage, allowing adequate samples to proceed to downstream testing while nondiagnostic cases are escalated to CNB when necessary, thereby reducing unnecessary use of core pathology resources.^34^ However, ROSE‐assisted FNA also depends on specialized personnel and thus may face similar capacity constraints.

The above‐mentioned differences reflect a pair of tensions that have not yet been clarified within the SVF system: There is a structural conflict between diagnostic sufficiency and value‐based allocation of medical resources. The default reliance of the current process on CNB may overlook the logic of *optimal value rather than maximum value* in the diagnostic path, specifically, how to achieve optimal resource utilization and ensure fair accessibility while meeting molecular detection requirements.

Under the trend of molecular oncology increasingly tending toward decentralized and individualized diagnosis, re‐examining the selection of initial diagnosis techniques under the SVF policy is no longer merely a matter of technical assessment but a policy priority to ensure the resilience and fairness of the system.

#### From sampling to action

The definition of diagnostic adequacy is evolving. Historically, it was centered around tissue volume and architectural integrity. Now, in the more value‐based era, it reorients toward actionable molecular yield, safety, and the time to treatment. Effective implementation of new sampling techniques depends on pragmatic material collection and handling. Under image guidance on lung lesions, clinicians typically obtain two to three FNA passes, with ROSE to confirm cellular adequacy. Adequate specimens are routed to cell‐block preparation or molecular fixation the same day, enabling prompt NGS. For example, using the Idylla platform (Biocartis) for rapid molecular testing directly on cytology smears or frozen pellets achieves results in just 2–3 hours, with high analytical accuracy (>94% across multiple target assays) and sensitivity to low variant allele frequencies, highlighting the feasibility of *same‐day molecular testing* in cytopathology practice.^35^ When cytology is borderline or nondiagnostic, pre‐arranged CNB pathways enable escalation within 48 hours, avoiding the scheduling drifts that commonly prolong diagnostic episodes. This structured approach optimizes tissue use, shifts effort from bulk processing to expert interpretation, and underpins the pre‐episode cost advantage observed in the Swedish case, for example.

### Future directions: Operationalizing the evidence

#### Data collection in routine

Carrying out priority pilot projects of FNA in regional hospitals is a key way to test its real‐world effectiveness. To ensure the success of this initiative and generate insights with promotional value, it is necessary to establish a systematic data collection mechanism covering multiple aspects such as clinical, operational, economic, and patient experience.

At the clinical and operational levels, the focus should be on (1) the time consumption of the diagnosis and treatment pathways; (2) the consistency of diagnostic accuracy with the routine diagnostic modality, including adequacy of samples based on tumor cell counts and the availability and use of ROSE; and (3) the incidence of complications. At the same time, it is necessary to track the proportion and reasons for upgrading to CNB or surgical biopsy because of insufficient samples and to evaluate the interval between re‐biopsies.

At the economic and system efficiency levels, it is necessary to examine the reimbursement level adjusted for complications, laboratory workload (including the preparation time of technicians and the number of sections/slides per case), and explore how to reinvest the resources saved by CNB in ROSE training or cell pathology capacity building, thereby forming a sustainable cost reallocation model.

At the patient level, indicators worth recording include the time required to travel to and from medical institutions, the number of days of leave for examinations, and the psychological burden quantified through anxiety scales then comparing these data with the cost structure of medical payers or suppliers. At the same time, case and institutional characteristics should also be collected, such as the size and location of the lesion, whether the patient is in an anticoagulant treatment state, the method of imaging guidance, as well as the level and type of medical institutions, to ensure the representativeness and generalizability of the research results.

Looking ahead, the continuous progress of molecular testing, especially the more sensitive NGS panels, and the rise of liquid biopsy technology are gradually reducing the reliance on the initial tissue volume. This trend suggests that minimally invasive sampling techniques, such as FNA, will have broader application scenarios and occupy a more core position in the future diagnostic ecosystem. Therefore, when designing the data‐collection plan for the pilot project, a forward‐looking perspective should be adopted. It is necessary not only to respond to the current decision‐making needs but also to provide a solid foundation for the in‐depth integration with emerging molecular detection technologies.

#### Research gaps

First, the microenvironment and spatial heterogeneity of the lesion remain unclear: There is a lack of comprehensive biobanks that associate CT imaging features (such as necrosis, fibrosis, and vascular distribution) with the adequacy of FNA and CNB samples in different NSCLC subtypes. The current unified operation plan should be applied to lesions with different biologic characteristics. In the future, it will be necessary to combine radiomics and genomics to establish a prospective paired biopsy library to improve and validate the indication rules, rather than relying on hypotheses.

Second, the mediating mechanism of survival benefits has not yet been fully clarified. Although FNA can accelerate the diagnosis and treatment process, the causal relation from biopsy selection to progression‐free or overall survival is influenced by disease stage, tumor biologic characteristics, and sample processing quality. Large‐scale mediation analyses need to be conducted in advanced patient cohorts using relevant diagnosis–treatment registration data to determine whether faster treatment pathways translate into lasting clinical outcome benefits.

Third, in the current economic assessment work, the burden on patients is mostly not explicitly considered. Anxiety, transportation costs, lost work hours, and out‐of‐pocket expenses can affect the rate of medical visits and treatment compliance, especially among rural populations. Validated, cross‐institutional biopsy burden measurement tools will help incorporate these factors along with clinical end points into the assessment system.

Finally, artificial intelligence–assisted cytology offers a new path to make up for the shortage of human resources in cellular pathology, but its scalability depends on a fair digitalization process and representative training data. Without regionally managed slice libraries and subsidized technical pathways, algorithms may exacerbate rather than alleviate medical inequality.

## CONCLUSION

As precision oncology increasingly relies on molecular information, this review points out that it is necessary to re‐examine the potential of FNA, from an auxiliary technique to an important component of the first‐line diagnostic strategy. In the context of CNB still being the *gold standard*, evidence demonstrates that FNA, supported by ROSE and in combination with molecular testing, achieves sampling adequacy comparable to CNB while lowering the complication risks. This approach shortens the diagnostic cycle and accelerates molecular subtyping and treatment decision‐making. FNA samples consistently provide sufficient DNA for multigene testing, achieving a high probability of consistency with surgical specimens in detecting key driver gene mutations. Its minimally invasive nature and reproducibility make it suitable for clinical scenarios that require repeated sampling, such as monitoring drug‐resistance evolution. Economic evaluation based on the Swedish SVF also indicates that prioritizing FNA saves approximately SEK 3889 per patient through reduced pathologic workload, lower complication costs, and optimized workflow.

In conclusion, this review argues for the standardized application of FNA in appropriate clinical settings, along with systematic cost‐effectiveness and long‐term, real‐world evaluation to establish a more efficient, equitable, and sustainable diagnosis strategy.

## AUTHOR CONTRIBUTIONS

**Jiayuan Guo:** Conceptualization; methodology; visualization; data curation; investigation; software; formal analysis; validation; writing—original draft; writing—review and editing; resources; project administration. **Bo Franzén:** Conceptualization; writing—review and editing; resources; project administration; supervision. **Rolf Lewensohn:** Conceptualization; writing—review and editing; resources; funding acquisition; project administration; supervision. **Martin Hysek:** Data curation; writing—review and editing; resources; project administration; supervision. **Linus Jönsson:** Conceptualization; writing—review and editing; resources; project administration; supervision.

## CONFLICT OF INTEREST STATEMENT

The authors declare no conflicts of interest.

## Supporting information

Supporting Information S1

## Data Availability

No new data sets were generated or analyzed. Extracted data and model assumptions referenced in the Swedish case study are available from the authors on reasonable request and/or in the Supporting Materials.
