# Supplementary material for: Economic value and clinical role of minimally invasive, biopsy‐based diagnostics in non‐small cell lung cancer
Source: Cancer Cytopathol. 2026 Sep 8;134(10):e70152. doi: 10.1002/cncy.70152 (PMC13552239; doi:10.1002/cncy.70152)
Supplement: Supplementary file 1 [file CNCY-134-0-s001.docx]

## **FIGURE S1: Full Referral & Triage Process**

## **SUPPORTING MATERIALS**

## **Cost-Benefit Calculation Model**

A2.1: To run this model, you need to collect the following data from the medical system you are in. It is recommended to collaborate with the radiology, pathology, finance, and quality control departments to obtain the most accurate information.

Notes to Appendix 2.1

Note 1: ROSE cost decomposition: The ROSE cost of 800 SEK is derived from 9 minutes of personnel time per case: 2 minutes for smear preparation, 2 minutes for staining, and 5 minutes for on‑site microscopic assessment. Based on an average Swedish pathologist/cytotechnologist hourly salary of approximately 5,330 SEK (including social benefits and overhead), this equates to 9/60 × 5,330 ≈ 800 SEK. Readers from other settings should substitute their own local salary data.

Note 2: CNB consumables: The CNB disposable gun cost is 299 SEK per unit. Additional consumables for CNB include one fine needle (62 SEK) for localisation, one Codan syringe (2 SEK), and one Lidocaine 20 ml ampoule (12 SEK). For FNA, consumables include two fine needles (2 × 62 = 124 SEK), two Codan syringes (2 × 2 = 4 SEK), and one Lidocaine 20 ml ampoule (12 SEK). These are already incorporated into the procedure costs (C_FNA and C_CNB) presented above.

Note 3: Institutional data: All "institutional" data are derived from Karolinska University Hospital accounting and registry records for the period 2023–2025. These are illustrative and intended for local calibration. Researchers applying this model elsewhere should substitute their own unit costs and complication/adequacy rates.

A2.2: Calculation Methodology

*Inputs&Parameter:*

$$C_{FNA}=FNA procedure cost$$

$$C_{CNB}=CNB procedure cost$$

$$C_{ROSE}=ROSE cost$$

$C_{slide}=cost per slide$

$$C_{pneum}=cost of pneumothorax management$$

$C_{bleed}=cost of hemorrhage management$

$p_{bleed}^{FNA},p_{bleed}^{CNB}=complication rates for FNA and CNB for hemorrhage$

$p_{pneum}^{FNA},p_{pneum}^{CNB}=complication rates for FNA and CNB for pneumothorax$

$S_{FNA,}S_{CNB}=first-pass adequacy rates (inadequacy = 1-s)$

*Formula:*

1. Pathology cost:

$Path_{CNB}=n slide of CNB \times C_{slide}$ ; $Path_{FNA}=n slide of FNA \times C_{slide}$

2. Expected complication cost:

$Co{mp}_{CNB}=p_{bleed}^{CNB}\times C_{bleed}+p_{pneum}^{CNB}\times C_{pneum}$ ; $Co{mp}_{FNA}=p_{bleed}^{FNA}\times C_{bleed}+p_{pneum}^{FNA}\times C_{pneum}$

3. Cost of Remedial Procedures:

$$B^{CNB}=C_{CNB}+Path_{CNB}+Co{mp}_{CNB}$$

Then $Rem^{CNB-first}=( 1-S_{CNB})\times B^{CNB}$; $Rem^{FNA-first}=( 1-S_{FNA})\times B^{CNB}$

4. Total Expected Cost:

$$Total^{CNB-first}=C_{CNB}+Path_{CNB}+Co{mp}_{CNB}+Rem^{CNB-first}$$

$$Total^{FNA-first}=C_{FNA}+C_{ROSE}+Path_{FNA}+Co{mp}_{FNA}+Rem^{FNA-first}$$

A2.3: Swedish Example (Illustrative Only)

$$C_{FNA}=5,000 SEK$$

$$C_{CNB}=8,135 SEK$$

$C_{ROSE}=800 SEKC_{slide}=120 SEK$

$C_{pneum}=15,000 SEKC_{bleed}=3,000 SEK$ $p_{bleed}^{FNA},p_{bleed}^{CNB}=2\%,5\%$ $p_{pneum}^{FNA},p_{pneum}^{CNB}=3\%,7\%$ $S_{FNA,}S_{CNB}=90\%,92\%$

Pathology cost:

$Path_{CNB}=12 \times120 = 1,440 SEK$ ; $Path_{FNA}=3\times120 = 360 SEK$

5. Expected complication cost:

$Co{mp}_{CNB}=(0.07 \times15,000) + (0.05 \times3,000) = 1,200 SEK$ $Co{mp}_{FNA}=(0.03 \times15,000) + (0.02 \times3,000) = 510 SEK$

6. Cost of Remedial Procedures:

$$B^{CNB}=8,135+1440+1,200=10,775 SEK$$

$Rem^{CNB-first}=0.08 \times10,775 = 862 SEK$;

$$Rem^{FNA-first}=0.10 \times10,775= 1,078 SEK$$

7. Total Expected Cost:

$$Total^{CNB-first}= (8,135 + 1,440 + 1,200) + 862= 11,637 SEK$$

$$Total^{FNA-first}=(5,000 + 800 + 360 + 510) + 1,078 = 7,748 SEK$$

Under these illustrative assumptions, an FNA-first strategy reduces the expected diagnostic cost per patient by 3,889 SEK (33% reduction) compared with CNB-first, primarily due to lower pathology workload, fewer complications, and reduced reliance on remedial procedures.

A2.4: Code Calculation for Figure 4

A2.5: Code Calculation for Figure 5

A2.6: Code for Figure 6 & Scenario analysis (sensitivity analysis)

It is not enough to only calculate the basic cases. To test robustness, a one-way deterministic sensitivity analysis was conducted and visualized in a tornado plot. It can visually show which parameter's fluctuation has the greatest impact on the total cost.

Scenario One: Fluctuations in the complication rate

Operation: Adjust the complication rates of FNA and CNB within the range of ±50%.

Objective: To test whether your conclusion is sensitive to changes in the complication rate. The complication rates may vary among different institutions and operators.

Scenario Two: Fluctuations in sample adequacy rate

Operation: Adjust the adequacy rates of FNA and CNB within the range of ±5% to 10% (for example, FNA: 85%-95%, CNB: 87%-97%).

Objective: To verify whether your conclusion still holds when there are reasonable fluctuations in sample quality.

Scenario Three: Hybrid Strategy (Real-world Model)

Operation: Suppose a certain proportion of patients (for example, 20%) undergo CNB directly for various reasons (such as suspected lymphoma, poor location). The "FNA first" strategy was only applied to the remaining 80% of patients and compared with the 100% "CNB first" strategy.

Objective: To make the model more realistic. No strategy is 100% applicable. This scenario calculates the net benefit of promoting the FNA-first strategy when some patients directly undergo CNB.
